# Genome-wide analysis and expression profile of the bZIP gene family in poplar

**DOI:** 10.1186/s12870-021-02879-w

**Published:** 2021-03-01

**Authors:** Kai Zhao, Song Chen, Wenjing Yao, Zihan Cheng, Boru Zhou, Tingbo Jiang

**Affiliations:** 1 State Key Laboratory of Tree Genetics and Breeding, Northeast Forestry University, 51 Hexing Road, Harbin, 150040 China; 2 Co-Innovation Center for Sustainable Forestry in Southern China/Bamboo Research Institute, Nanjing Forestry University, 159 Longpan Road, Nanjing, 210037 China

**Keywords:** Poplar bZIP gene family, Tissue-differential expression, Salt stress, Co-expression analysis

## Abstract

**Background:**

The bZIP gene family, which is widely present in plants, participates in varied biological processes including growth and development and stress responses. How do the genes regulate such biological processes? Systems biology is powerful for mechanistic understanding of gene functions. However, such studies have not yet been reported in poplar.

**Results:**

In this study, we identified 86 poplar bZIP transcription factors and described their conserved domains. According to the results of phylogenetic tree, we divided these members into 12 groups with specific gene structures and motif compositions. The corresponding genes that harbor a large number of segmental duplication events are unevenly distributed on the 17 poplar chromosomes. In addition, we further examined collinearity between these genes and the related genes from six other species. Evidence from transcriptomic data indicated that the bZIP genes in poplar displayed different expression patterns in roots, stems, and leaves. Furthermore, we identified 45 bZIP genes that respond to salt stress in the three tissues. We performed co-expression analysis on the representative genes, followed by gene set enrichment analysis. The results demonstrated that tissue differentially expressed genes, especially the co-expressing genes, are mainly involved in secondary metabolic and secondary metabolite biosynthetic processes. However, salt stress responsive genes and their co-expressing genes mainly participate in the regulation of metal ion transport, and methionine biosynthetic.

**Conclusions:**

Using comparative genomics and systems biology approaches, we, for the first time, systematically explore the structures and functions of the bZIP gene family in poplar. It appears that the bZIP gene family plays significant roles in regulation of poplar development and growth and salt stress responses through differential gene networks or biological processes. These findings provide the foundation for genetic breeding by engineering target regulators and corresponding gene networks into poplar lines.

**Supplementary Information:**

The online version contains supplementary material available at 10.1186/s12870-021-02879-w.

## Background

The basic leucine zipper (bZIP) represents a super gene family that encodes transcription factors. This gene family is widely distributed in eukaryotes. The bZIP proteins, which are defined by the conserved bZIP domain [1, 2], play significant roles in the regulation of various biological processes, such as plant growth and development and salt stress responses.

Transcription factor proteins coded by the bZIP gene family contain a highly conserved bZIP domain. The structure is composed of 60–80 amino acids, including a basic DNA binding region and adjacent leucine zipper [2]. The binding region contains nuclear localization signals and an N-X_7_-R/K motif with constant precise intervals to contact target DNA [3]. The leucine zipper region is composed of heptad repeats of leucine or other large hydrophobic amino acids, and the number of repeats in different genes may vary greatly [3, 4]. Leucine is located at the seventh amino acid position of the heptapeptide sequence and may be replaced by isoleucine, valine, phenylalanine or methionine [3]. The bZIP proteins usually function by forming dimers through the leucine zipper [4]. Plant bZIP transcription factors have a binding preference for ACGT core sequences, such as A-box (TACGTA), C-box (GACGTC), and G-box (CACGTG). In addition, they also bind to other DNA sequence motifs [3, 4]. The outer flanks of the core element regulate specificity of protein-DNA interactions [3]. Previous studies have indicated that segmental genome duplications and whole-genome duplication events may explain expansion of the bZIP gene family [2, 5]. Regarding classification, researchers initially divided the bZIP gene family members into 10 groups in Arabidopsis, based on common domains [3]. Then the Arabidopsis bZIP genes were updated and further divided into 13 groups (A-M) [4].

The bZIP gene family plays important roles in biological processes, such as growth and development, maturation of flowers, and stress responses in plants. The Arabidopsis *bZIP11* gene impacts root development by linking low-energy signals to auxin-mediated control of primary root growth [6]. *HY5* encodes a bZIP protein, which is involved in the regulation of Arabidopsis root and hypocotyl development [7]. Overexpression of *ZmbZIP4* in maize leads to an increase in the number of lateral roots, longer primary roots, and an improved root system [8]. The bZIP proteins also regulate plant responses to abiotic stresses, such as salt stress. Over-expression of *SlAREB* gene in tomato can improve plant tolerance to water deficiency and salt stress [9]. Similar results were observed for the *GhABF2* gene in Arabidopsis and cotton [10], as well as for *GmbZIP2* in transgenic soybean [11].

Studies on the bZIP gene family in poplar have focused only on its functions in root development and drought resistance. For example, poplar *PtabZIP1L* is mainly expressed in roots and can mediate the development of lateral roots and drought resistance by regulating various metabolic pathways [12]. Poplar *bZIP53* is inducible in gene expression by salt stress and negatively regulates the development of adventitious roots [13]. The poplar *AREB1* can regulate drought responses and tolerance of *Populus trichocarpa* by affecting histone acetylation [14]. The comprehensive analysis and characterization of the poplar bZIP gene family and the screening of tissue differentially expressed genes (DEGs) and salt stress response genes using transcriptome sequencing can provide an important reference for gene function researches and genetic engineering breeding. In addition, using bioinformatics methods to reveal the biological processes that genes may participate in will help to analyze the regulatory mechanism of genes. In the present study, we performed systematic investigation on the bZIP genes in poplar, including identification of gene family members; protein sequence analyses and phylogenetic relationships; chromosomal distribution of the genes; genomic tandem duplications and segmental duplications; and collinearity analysis across species. Furthermore, based on transcriptome profiling data, we also explored differential expression patterns of the bZIP genes across different tissues, and their responses to salt stress. Finally, we performed gene co-expression and network analyses on the key genes, followed by gene set enrichment analyses. Our systems biology approach has shed light on differential gene networks or biological pathways associated with varied biological processes.

## Results

### Identification and characterization of the bZIP transcription factor family in poplar

**Fig. 1:**
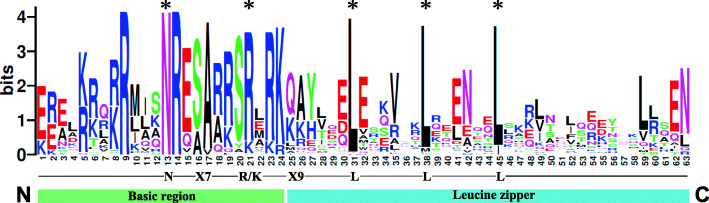
Visualization of multiple sequence alignment of the poplar bZIP family DNA binding domains. The total height of the letter piles at each position indicates the conservation of the sequence at that position (measured in bits). The height of a single letter in the letter piles represents the relative frequency of the corresponding amino acid at that position

In this study, we identified 86 proteins from the bZIP family, by use of HMMER analysis (E-value < 1 × 10^− 5^). We then used Pfam and SMART databases to verify the results [15–17]. Evidence from verification by Pfam and SMART indicated that the 86 poplar proteins shared the bZIP domain, which is congruent to our predictions. Thus we extracted the amino acid sequences of the bZIP conserved domain from each member and performed multiple sequence alignment [18]. The results are visualized in Fig. 1. The bZIP domain is composed of a basic DNA-binding region and an adjacent leucine zipper structure. The basic region contains an invariable N-X_7_-R/K motif, while the ZIP domain is composed of heptapeptide repeat of Leucine (L) or related hydrophobic amino acid. The highly conserved leucine residues are occasionally replaced by isoleucine, methionine, etc. (Fig. 1). Compared to the previous studies in Arabidopsis [3, 4], our results are congruent to those.

### Phylogenetic tree and sequence structure analysis

**Fig. 2:**
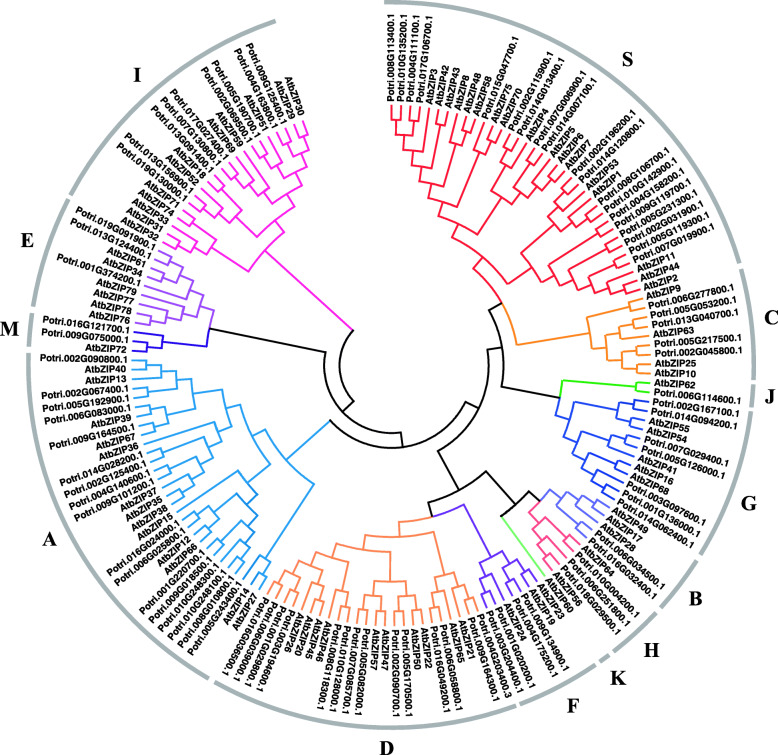
Dendrogram of poplar and Arabidopsis bZIP members. The dendrogram was drew by MEGA7 with the Maximum Likelihood method and JTT + G + F model. Different groups are marked with different colors. The groups were named with letters representing some of their important members (A for ABF/AREB/ABI5, C for CPRF2-like, G for GBF, H for HY5), protein size (B for big, S for small), or alphabetically

To explore the evolutionary relationships and classification of the bZIP family, we constructed a phylogenetic tree, using the entire amino acid sequences of each member from both poplar and Arabidopsis. The Arabidopsis bZIP family members are divided into 13 groups using letters representing some of their important members (A for ABF/AREB/ABI5, C for CPRF2-like, G for GBF, H for HY5), protein size (B for big, S for small), or alphabetically [3, 4]. As shown in Fig. 2, we divided the poplar bZIP proteins into 12 groups, based on previous studies in *Arabidopsis thaliana*. The size of the 12 groups varies. The three largest groups have 19 (S group), 17 (A group), and 14 (D group) members, while groups J, B, M, and K have only 1, 2, 2, and 0 members, respectively.

**Fig. 3:**
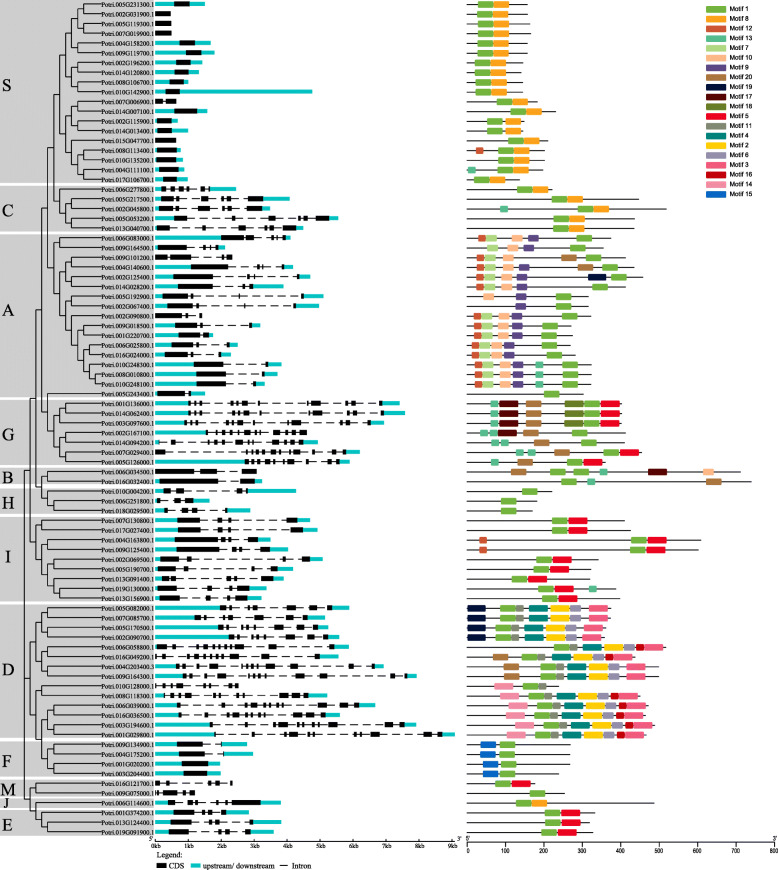
DNA structures and protein motifs of the bZIP gene family in poplar. **a** Gene structures. Exons and 5′ UTR/3′ UTR are displayed using black and cyan bars. Black dotted lines denote introns. **b** Protein motifs in the bZIP members. The colorful boxes delineate different motifs. The clustering is performed according to the results of phylogenetic analysis

In addition, to explore the sequence structure of the poplar bZIP family, we analyzed the intron/exon structure and motif composition of each member (Fig. 3). As expected, members of the same group, especially some close members, share similar gene structures. For example, out of the 19 members in the S group, 18 contain only one exon. All members in group C harbor 6 exons and 5 introns. Out of the 7 members in group G, 6 have 12 exons and 11 introns. Interestingly, many members with a closer relationship also share similar exon lengths (Fig. 3).

Using MEME [19], we found a total of 20 conserved motifs (Fig. 3). Through the annotations of SMART and Pfam databases, we found that motif 1 is a bZIP domain, motif 2 and 4 are DOG1, and other motifs have no specific annotation information (Supplemental Table 1). As expected, all of the poplar bZIP members share the motif 1. In contrast, motifs 2, 3, 4, and 6 exist only in the majority of members in group D. Similarly, motifs 7 and 9 occur only in Group A. Motif 8 presents only in groups S, C, and J. Motif 10 rests only in group A and B. Motif 11 is shared only by all members in group D. Motif 14 exists only in the six members of group D. Motif 15 exists only in all members of group F. Motif 16 occurs only in the 7 members of group D. Motif 17 exists only in the 5 members of group G and B. Motif 18 presents only in the three members of group G. Many motifs exist in specific groups, which might be related to specific biological functions.

### Chromosomal location and collinearity analysis of the bZIP gene family in poplar

**Fig. 4:**
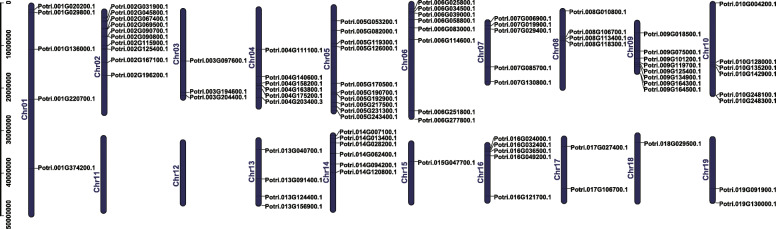
Chromosome distribution of poplar bZIP genes. Chr01–19 represent chromosome numbers 01–19

Using poplar genome annotation information and TBtools [20, 21], we visualized the chromosomal distribution of the bZIP gene family. Results from Fig. 4 indicated that the 86 bZIP genes are unevenly distributed on the 17 chromosomes, and the number of genes on each chromosome is irrelevant to chromosome size. For example, the largest chromosome (Chr 1) contains only 5 genes, while the smallest chromosome (Chr 9) contains 8 bZIP genes. No genes were located on chromosomes 11 or 12.

**Fig. 5:**
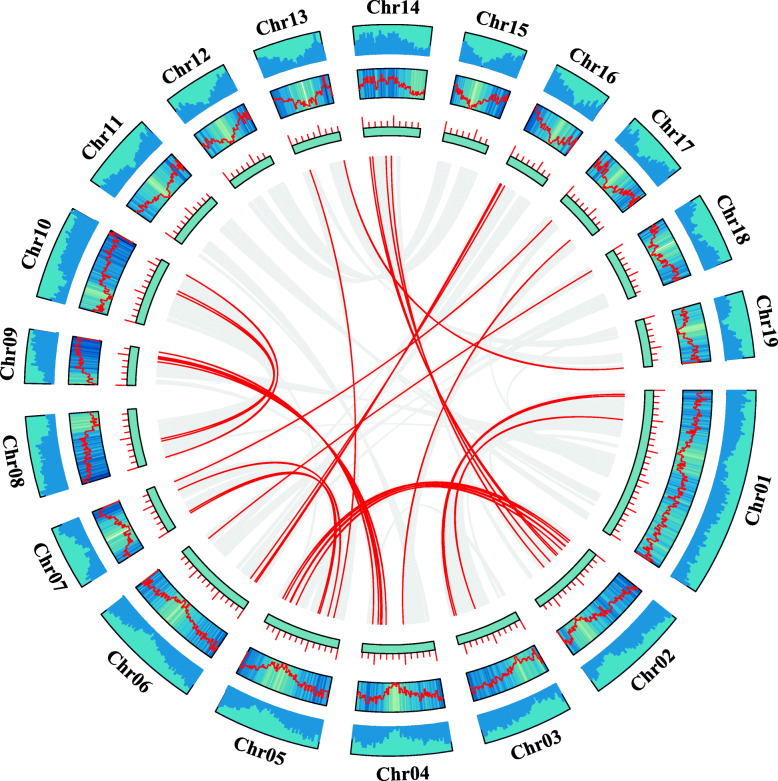
Collinearity analysis of the bZIP gene family in poplar. Chromosomes 01–19 are represented by blue rectangles. The lines, heatmaps, and histograms along the rectangles represent gene density on the chromosomes. The gray lines indicate synteny blocks in the poplar genome, while the red lines between chromosomes delineate segmental duplicated gene pairs

Subsequently, using TBtools with the Multiple Collinearity Scan toolkit (MCScanX) method [21, 22], we analyzed the tandem duplication events among the genes. Interestingly, no tandem duplication events were identified. Then, we used TBtools with the BLASTP and MCScanX methods to analyze the segmental duplication events [21, 22]. Our results are shown in Fig. 5 and Supplemental Table 2. We identified a total of 31 gene pairs with segmental duplication events, which occurred on 16 of the 19 chromosomes. These lines of evidence suggest that segmental duplication events are the main driving force for the diversity of the bZIP genes in poplar.

**Fig. 6:**
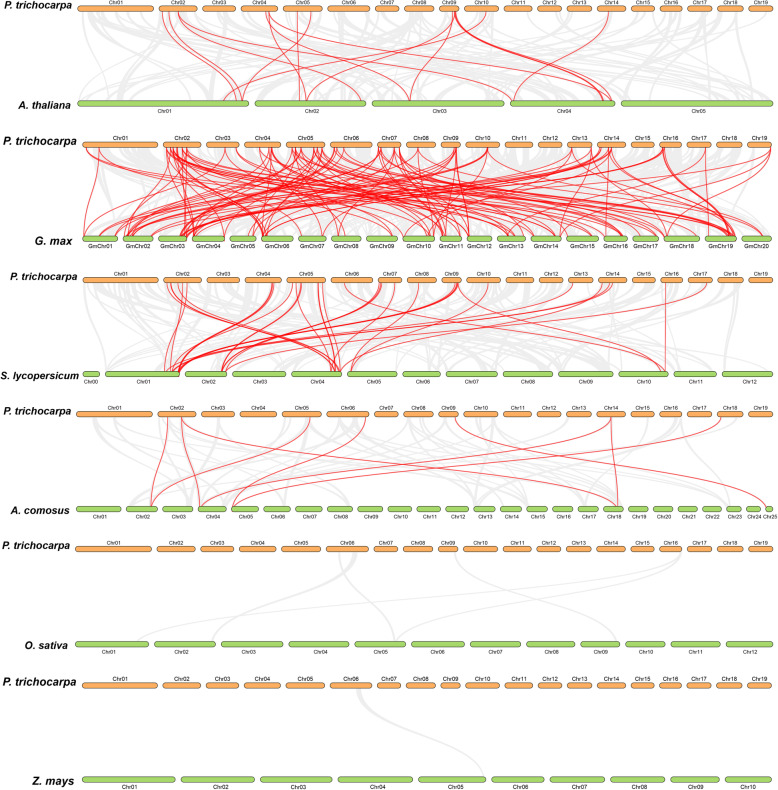
Synteny analysis of the bZIP genes between poplar and six other plant species. The gray lines indicate gene blocks in poplar that are orthologous to the other genomes. The red lines delineate the syntenic bZIP gene pairs

Furthermore, we also explored the collinearity relationships between the poplar bZIP genes and related genes from six representative species, including three eudicots (*Arabidopsis thaliana*, *Glycine max*, and *Solanum lycopersicum*) and three monocots (*Oryza sativa*, *Zea mays*, and *Ananas comosus*), to explore orthologs (Fig. 6, Supplemental Table 3). A total of 55 poplar genes have collinearity relationships with 11 Arabidopsis genes, 74 soybean genes, 20 tomato genes, and 5 pineapple genes. However, there is no such relationship between poplar genes and rice or maize genes (Supplemental Table 3). The number of orthologous gene pairs is 16 between poplar and Arabidopsis, 127 between poplar and soybean, 34 between poplar and tomato, and 9 between poplar and pineapple. However, no such gene pairs were identified between poplar and rice, and between poplar and maize. These may be explained by the closer phylogenetic relationships among the dicots relative to monocots.

It is worth noting that there was great collinearity between the poplar genes and the soybean genes, than those found with the other five species. This may be related to the fact that both poplar and soybean belong to fabids. In addition, we found that a large number of the poplar bZIP genes have collinearity relationships with three to four soybean genes, suggesting that these genes may play important roles in the evolution of the gene family. In addition, we also found that two poplar bZIP genes (*Potri.006G251800.1* and *Potri.018G029500.1*) are collinear with pineapple genes. However, there is no such relationship with the genes from the other five plants, suggesting that these genes have been retained in pineapple and poplar and have been lost in the rest of the plants analyzed.

We also used TBtools to calculate the Non-synonymous (Ka) / synonymous (Ks) ratios for each gene pair, to explore the evolutionary constraints of the poplar bZIP genes [21]. The Ka/Ks ratios of both segmental duplication and collinearity gene pairs are less than 1. This means that the poplar bZIP gene family might have experienced strong purifying selection pressure in the process of evolution.

### Tissue-differential gene expression of poplar bZIP genes

**Fig. 7:**
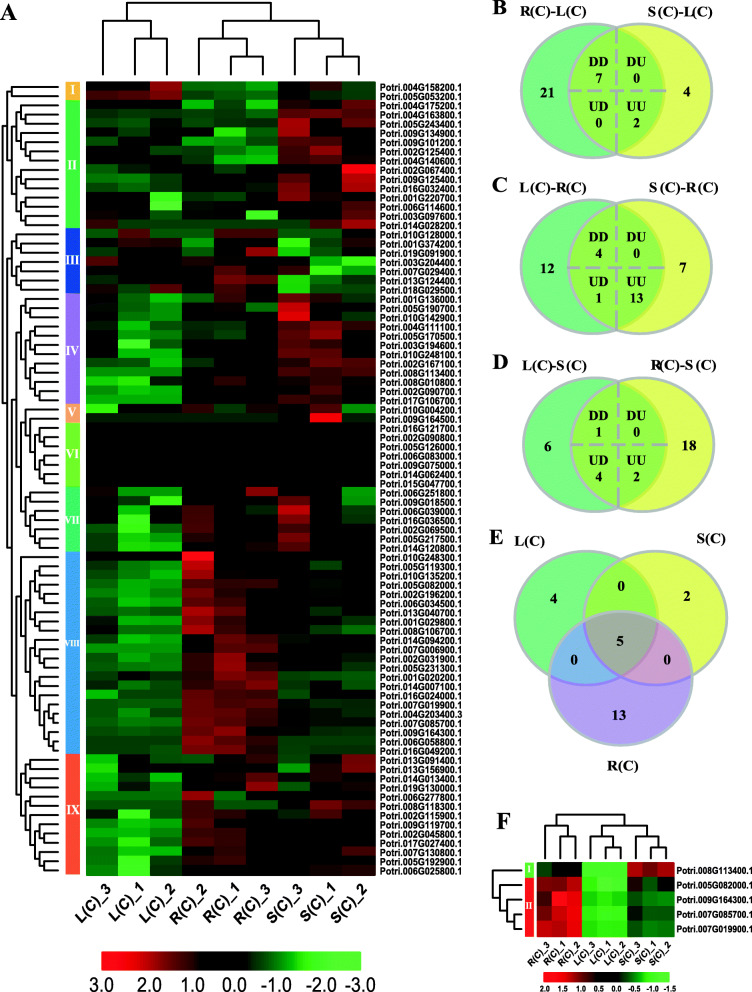
Differentially expressed bZIP genes over tissues in poplar. **a** Heatmap of poplar bZIP gene expression without treatment. **b-d** Number of tissue differentially expressed genes displaying distinct and shared expression between pairs of tissues without treatment. The numbers in the shared part of each figure are DEGs in one tissue relative to the other two tissues. DD and UU represent down- and up-regulated in the two comparisons. DU stands for down-regulation in the left comparison and up-regulation in the right comparison. UD is opposite to DU. **e** Comparisons of the shared genes from B to D. The shared five genes were differentially expressed in any tissue relative to the other two tissues. **f** Heatmap of five genes shared in panel E. L(C), S(C), and R(C) represent leaves, stems, and roots without treatment

To explore expression patterns of the poplar bZIP gene family in different tissues, we used transcriptome profiling data from RNA-Seq to analyze the bZIP gene expression in poplar roots, stems, and leaves. As shown in Fig. 7a, we divided these genes into 9 groups with specific patterns. For example, genes in groups 2 and 4 are highly expressed in stems. In contrast, those of group 8 have higher expression levels on roots.

We then used the DESeq method to identify DEGs across the tissues [23]. Our results indicated that there were 30 DEGs between leaves and roots (8 and 22 down- and up-regulated in roots, respectively), 13 between leaves and stems (5 and 8 down- and up-regulated in stems, respectively), and 25 between roots and stems (15 and 10 down- and up-regulated in stems, respectively) (Supplemental Data 1). Subsequently, we identified genes that are differentially expressed in one tissue relative to the other two tissues. As shown in Fig. 7b-d and Supplemental Data 1, the majority of DEGs (18) were identified in roots. Among them, 4 and 13 genes were down- and up-regulated in roots relative to the other two tissues, respectively. And there is also a gene that was up-regulated in roots relative to leaves, but was down-regulated in roots relative to stems. Similarly, we identified 9 DEGs in the leaves. Among them, respective 7 and 2 genes were down- and up-regulated in leaves compared to stems and roots. Seven DEGs were identified in the stems. Among them, one gene was down-regulated and 2 genes were up-regulated in stems compared to leaves and roots. Furthermore, four genes were up-regulated in stems relative to leaves, but were down-regulated in stems relative to roots. Finally, we compared the three sets of genes identified above, and obtained 5 shared genes, which were differentially expressed in any tissue relative to the other two tissues (Fig. 7e). We then drew a heatmap, based on the expression data of these 5 genes (Fig. 7f). It appears clear tissue-specific expression patterns; that is, one gene showed high, moderate, or low expressed in stems, roots, and leaves, respectively. However, the rest of the genes displayed a different pattern (Fig. 7f).

### Gene expression in response to salt stress in poplar

**Fig. 8:**
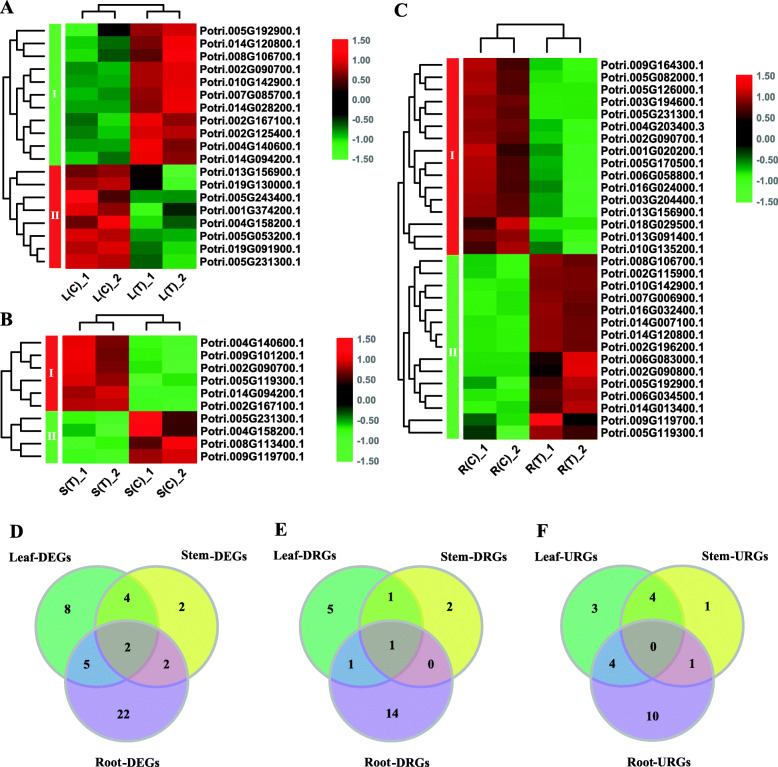
Differentially expressed bZIP genes in response to salt stress in poplar. **a**-**c** Heatmaps of the bZIP genes in response to salt stress in leaves, stems, and roots. L, S, and R denote leaves, stems, and roots, respectively. T and C represent with and without salt treatment, respectively. **d**-**f** Venn diagrams of DEGs, down-regulated DEGs (DRGs), and up-regulated DEGs (URGs) in response to salt stress in various tissues

To explore expression patterns of the bZIP gene family in response to salt stress, we analyzed expression changes of the responsive genes before and after salt stress, based on the RNA-Seq data. As shown in Fig. 8a-c and Supplemental Data 2, we identified the majority of DEGs in roots (16 and 15 down- and up-regulated, respectively), followed by 19 genes in leaves (8 and 11), and by 10 genes in stems (4 and 6). We then compared the three groups of genes and drew Venn diagrams (Fig. 8d-f). As shown in Fig. 8d, the majority of genes (22) responsive to salt stress were found to be specific to roots, followed by 8 genes to leaves, and by 2 genes to stems. In addition, 5 genes were responsive to the salt stress in both leaves and roots (leaf-root), 4 genes in leaf-stem, and 2 genes in root-stem. Among them, 2 genes overlap between the three groups of DEGs (Fig. 8d). Comparisons of down- and up-regulated DEGs are shown in Fig. 8e, f.

### Validation of DEGs by qRT-PCR

**Fig. 9:**
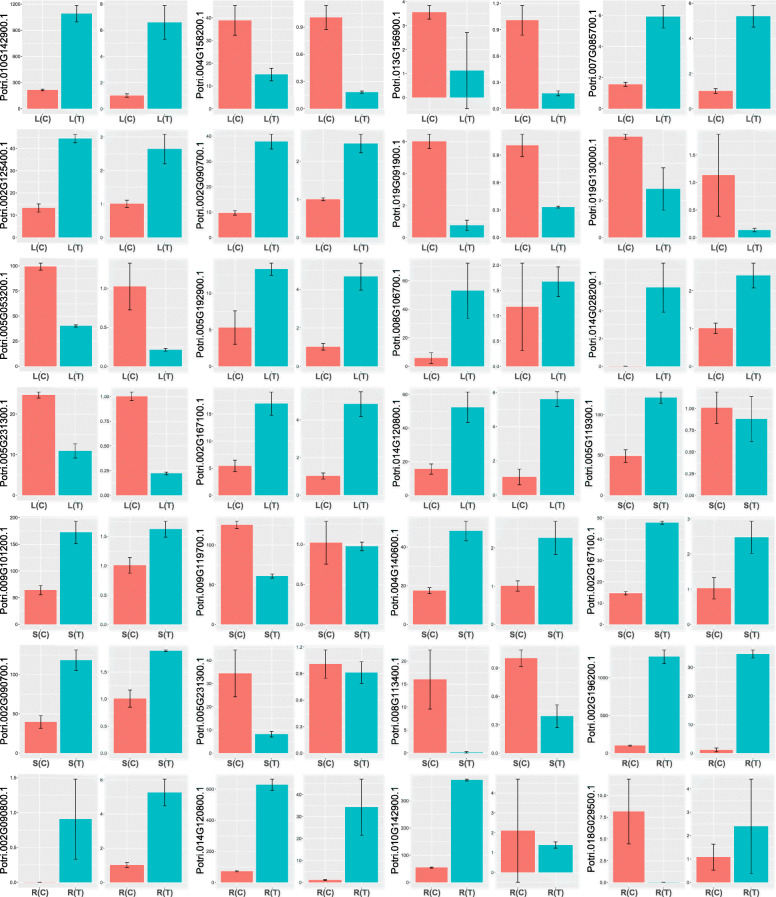
Gene expression levels based on RNA-Seq and qRT-PCR. L, S, and R denote leaves, stems, and roots, respectively. T and C represent with and without salt treatment, respectively. Error bars are standard deviations from the biologic replicates

To verify the results of RNA-Seq, we used qRT-PCR to quantify expression levels of the 23 DEGs in roots, stems and leaves, before and after the salt stress. As shown in Fig. 9, results from RNA-Seq and qRT-PCR are congruent.

### Gene co-expression analysis

**Fig. 10:**
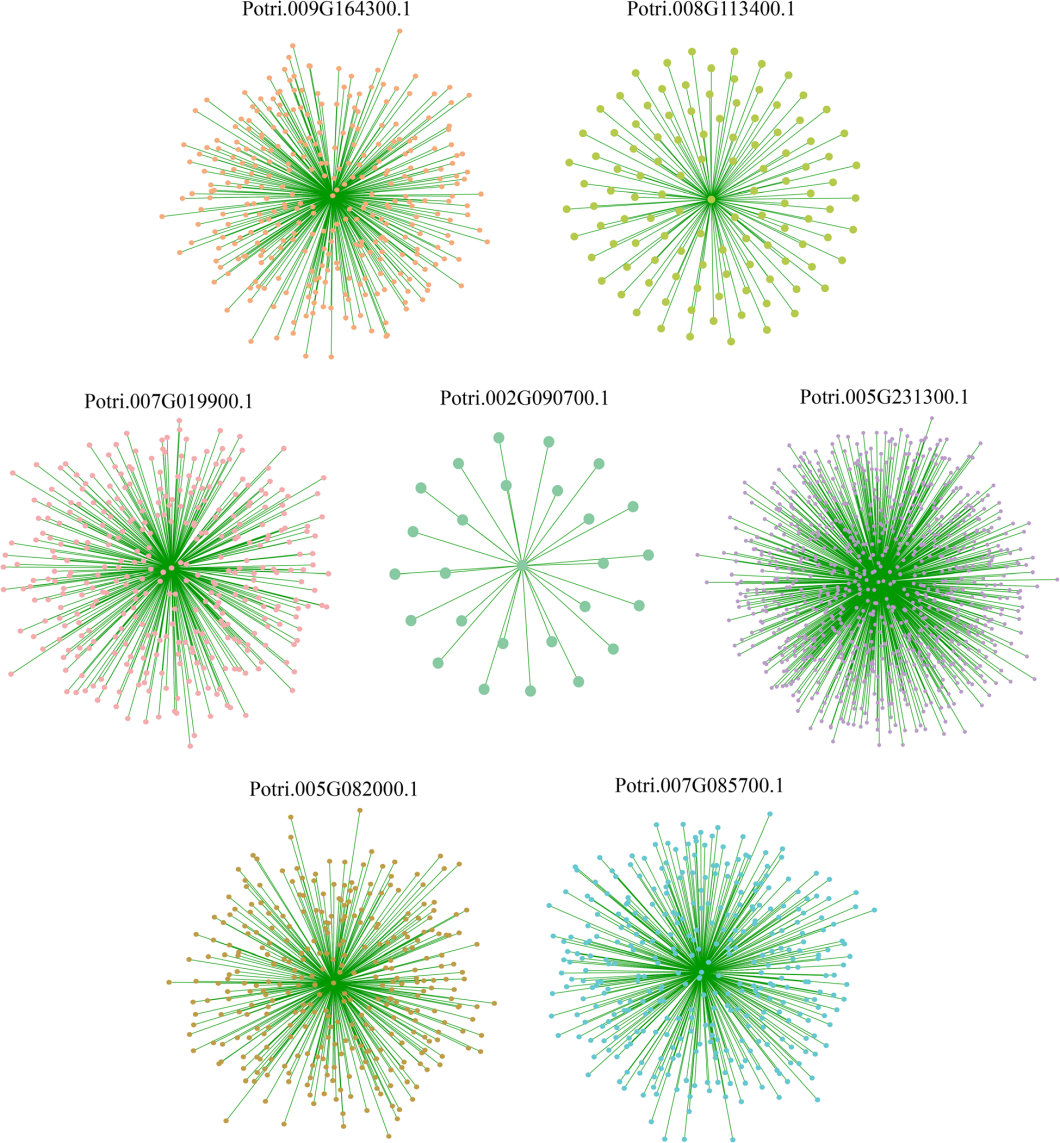
TF-centered co-expression network of five tissue differentially expressed genes and two salt stress response genes. Dots represent genes, and lines indicate that they have co-expression relationship

Co-expression analysis can help find genes with similar expression patterns. These genes may be closely co-regulated, tightly related in function, or members involved in the same signaling pathway or physiological process. In this study, we used the Weighted Correlation Network Analysis (WGCNA) method and the RNA-Seq data of 21 samples to construct a co-expression network centered on the 5 tissue-differential expressed genes and the 2 salt responsive genes described above [24]. As shown in Fig. 10 and Supplemental Data 3, we obtained a total of 7 co-expression networks. Among them, the network centered on *Potri.005G231300.1* is the largest (855 genes). In contrast, the network centered on *Potri.002G090700.1* is the smallest (27 genes).

**Fig. 11:**
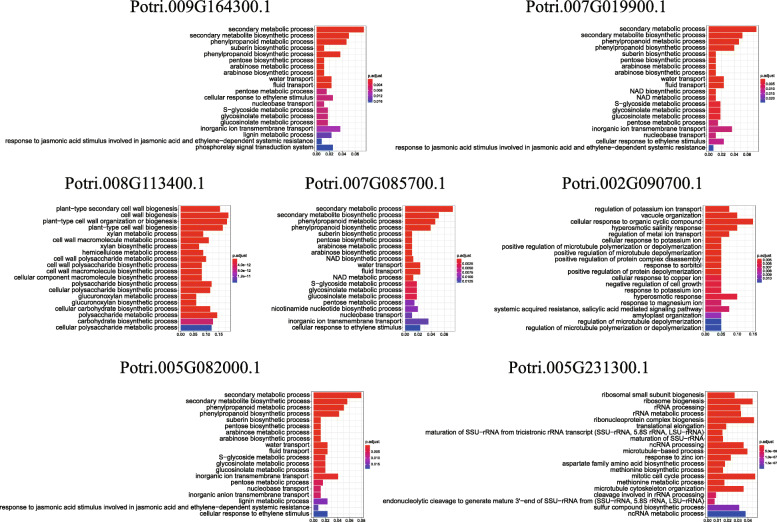
Go enrichment analysis of seven co-expressed gene sets

To explore the biological processes that these genes may participate in, we then conducted gene set enrichment analysis on the 7 sets of co-expressed genes identified above (Fig. 11). Four of the five tissue-differential expressed genes (*Potri.005G082000.1*, *Potri.007G085700.1*, *Potri.007G019900.1*, *Potri.009G164300.1*), share 17 significantly enriched GO Terms. The shared GO Terms include secondary metabolic process, secondary metabolite biosynthetic process, phenylpropanoid metabolic process, and phenylpropanoid biosynthetic process. This suggests that the four genes may play important roles in regulation of poplar growth and development and stress responses. Interestingly, the four genes have the same expression pattern across the tissues (Fig. 7f).

Other genes involved in tissue differential expression and the co-expressed gene network are significantly enriched in cell wall biogenesis, cell wall macromolecule biosynthetic process, xylan metabolic process, xylan biosynthetic process, hemicellulose metabolic process, and other biological processes, suggesting that they may be related to formation of cell walls.

Regarding the genes that respond to salt stress in the root, stem and leaf tissues, *Potri.002G090700.1* and its co-expressed gene network are significantly enriched in GO terms, such as regulation of potassium ion transport, hyperosmotic salinity response, regulation of metal ion transport, and hyperosmotic response. It is suspected that these genes may respond to salt or osmotic stresses by regulation of ion balance. *Potri.005G231300.1* and the genes in its network are significantly enriched in GO terms, such as methionine biosynthetic process and methionine metabolic process. The S-Adenosyl-L-methionine synthetase (SAMS) in plants can catalyze the reaction of methionine and ATP to produce S-Adenosyl-L-methionine, which is a key enzyme that regulates the methionine cycle. Previous studies have shown that *SAMS* is a key gene for organisms to resist adversity and stress [25]. Therefore, we speculate that these genes might be involved in the processes of methionine synthesis and metabolism, which are related to stress resistance.

## Discussion

The bZIP transcription factors are found in the plant kingdom, which play important roles in regulating growth and development and responses to biotic and abiotic stresses [4, 26]. Previous studies on poplar were constrained on only a few bZIP genes, which regulate root development and drought stress. Thus systematic studies on the poplar bZIP gene family have not been reported. In this study, we used strict standards to identify 86 bZIP genes from poplar. Then we extracted the bZIP protein domain sequences of the members. Evidence from multiple sequence alignment indicated that both poplar and Arabidopsis share the same bZIP domain. Compared to Arabidopsis [3, 4], the poplar bZIP domains also consist of a DNA binding region containing the N-X_7_-R/K motif and a leucine zipper structure. We then divided these 86 members into 12 groups, based on similarity of their protein sequences. It is worth noting that many motifs exist in specific groups. Given that the bZIP transcription factors have varied functions, these motifs might perform specific functions. This phenomenon deserves further studies.

Previous studies indicated that in the evolutionary process gene families usually undergo tandem duplication or large-scale segmental duplication, to maintain a large size of each family [27]. Unlike birch, which has not undergo recent whole genome duplication [28], poplar has undergone at least three rounds of whole-genome duplication, followed by multiple segmental duplication, tandem duplication and transposition events [20, 29]. Since the bZIP gene family is a relatively large one, thus we analyzed both the tandem duplication events and the segmental duplication events. Interesting, we found that there were no tandem duplication events in the poplar bZIP genes. However, a large number of segmental duplication events occurred, which is consistent with previous studies in rice [2]. These results indicate that segmental duplication events play an important role in the expansion of the bZIP gene family. Furthermore, we analyzed the collinearity between the poplar bZIP genes and the counterparts from three eudicots and three monocots. The results demonstrated that there were significantly more collinearity gene pairs between poplar and eudicots than between poplar and monocots. Species with relatively close evolutionary relationships, such as poplar and soybean, appeared to have more collinear gene pairs. We also identified many poplar bZIP genes that existed either in multiple such gene pairs or only in the collinearity with monocots. The calculated values of the Ka/Ks ratios for all gene pairs were less than 1, suggesting that these genes might have experienced strong purifying selection pressure in evolution.

We explored expression patterns of the bZIP genes in a tissue-specific fashion. We have identified many DEGs between tissues, of which five genes are differentially expressed in any two of the three tissues. These genes showed low expression in leaves and high expression in roots or stems. We then mapped these genes to the Arabidopsis genome to understand their possible functions (Supplemental Table 4) [30, 31]. The best match for *Potri.005G082000.1* and *Potri.007G085700.1* is *AT5G65210.1*, which is an essential cofactor in BOP-dependent regulation of development in Arabidopsis [29]. *AT1G08320.1* is the counterpart for *Potri.009G164300.1*, which is involved in the Arabidopsis anther development [32]. *AT1G75390.1* is the best match for *Potri.007G019900.1*, which affects the germination process of seeds [33]. Taken together, these tissue-specific DEGs are likely to play important roles in poplar growth and development.

Since little is known in poplar about the functions of the bZIP genes in regulation of salt responses, we explored their expression patterns in response to salt stress. Comparing the expression data from samples both before and after salt stress, we identified a total of 45 genes that respond to salt stress in roots, stems, and leaves. We also mapped them to the Arabidopsis genome (Supplemental Table 4) [30, 31]. The homologous genes are involved in growth and development of Arabidopsis, as well as in responses to abiotic stresses. For example, *AT2G40950.1*, a homologous gene of *Potri.006G034500.1* and *Potri.016G032400.1*, is a transcription factor regulating cellular responses to salinity through osmotic stress adjustment [34]. The best matches for *Potri.001G020200.1* (*AT4G35040.1*) and for *Potri.003G204400.1* (*AT2G16770.1*) were reported to regulate adaptation of Arabidopsis to zinc deficiency [35]. *AT1G45249.1*, the counterpart for *Potri.009G101200.1*, *Potri.014G028200.1*, and *Potri.002G125400.1*, were able to regulate ABRE-dependent ABA signaling pathway related to drought stress tolerance [36].

To understand their function, we performed co-expression based gene network analysis, focusing on the seven key genes we identified, followed by gene set enrichment analysis. Our results from gene ontology analyses indicated that the DEGs across tissues and their corresponding network genes are enriched in secondary metabolic process, and secondary metabolite biosynthetic process. However, the salt-inducible genes and their corresponding network genes are enriched in regulation of metal ion transport and methionine biosynthetic processes. These suggest that different regulators and regulated gene networks play important roles in specific biological functions.

## Conclusions

In this study, we identified 86 bZIP gene family members in poplar and characterized their conserved bZIP domains. We then conducted systematic analyses of the gene family. On the basis of the phylogenetic analyses, these members can be divided into 12 groups and each group has specific gene structure and motif composition. The bZIP genes are unevenly distributed on the 17 chromosomes of poplar. It is worth noting that no tandem duplication events were identified between poplar bZIP genes, however, we detected a large number of segmental duplication events, which suggest that segmental duplication events are the main driving force for the evolution of the bZIP gene family in poplar. In addition, we investigated the collinearity relationship between the poplar bZIP genes and the homologous genes from six representative species. These will benefit future comparative gene functions studies. Furthermore, we identified tissue-specific gene expression patterns, as well as salt-inducible gene expression patterns. On the basis of the seven key regulators and their corresponding gene networks, differential regulators and corresponding gene networks have been found to associate with varied biological processes.

## Methods

### Identification of poplar bZIP transcription factors and their conserved domains

The amino acid sequences of all poplar proteins were downloaded from the Phytozome database [20, 30], and that of bZIP_1 (PF00170) from the Pfam database [17]. First, we used hmmsearch (http://www.hmmer.org/) with bZIP_1 to search the poplar amino acid sequences, with a threshold of E-value < 1 × 10^− 5^. After obtaining the candidate genes, we applied Pfam and SMART databases to further verify our results [15–17]. Then, we extracted sequences of the conserved domains from the poplar bZIP proteins identified. Finally, we used ClustalX 1.83 and WebLogo for multiple sequence alignment and visualization, respectively [18, 37].

### Classification and sequence analysis on the bZIP members

The amino acid sequences of both poplar and Arabidopsis family members were downloaded from the Phytozome database [20, 30, 31]. Members of the Arabidopsis bZIP gene family were identified from previous studies [4].

We used the MEGA 7 with Maximum Likelihood method and the best model selected by SMS to construct a phylogenetic tree [38, 39]. Classification of the poplar bZIP protein family refers to previous studies in Arabidopsis [4].

To analyze structure of the bZIP gene family, we downloaded the genome sequences and coding sequences of the genes from the Phytozome database [20, 30]. We then used the Gene Structure Display Server to draw gene structure diagrams [40]. The order and grouping of genes in the gene structure diagrams refer to the results of the phylogenetic tree. Using MEME, we identified conserved motifs contained in the bZIP gene family [19]. Finally, we imported the generated file into TBtools for visualization [21]. Motif annotation information comes from the SMART and Pfam databases [15–17].

### Chromosome distribution and collinearity analysis

Using the TBtools and the poplar genome data downloaded from the Phytozome database [20, 21, 30], we visualized the chromosomal distribution of the bZIP genes in poplar. In addition, using the TBtools with MCScanX, we analyzed the tandem duplication events of the bZIP gene family [21, 22]. Similarly, using the TBtools with MCScanX and BLASTP methods, we investigated segmental duplication events and the collinearity relationship for gene pairs from different species [21, 22]. Ka and Ks substitutions between gene pairs were also calculated, by use of the TBtools [21].

### Plant materials and gene expression analysis

The plant materials used in this study were di-haploid *Populus simonii × Populus nigra* seedlings from a wild-type clone growing in the experimental forest of Northeast Forestry University. Using the RNA-Seq data described in our previous study [41], we explored tissue differential expression patterns of the bZIP genes. To investigate expression patterns of the genes in response to salt stress, we used the RNA-Seq data collected from root, stem, and leaf tissues under treatment with 0 or 150 mM NaCl for 24 h [42]. All of the samples have 10X sequencing depth. Using the DESeq package in R, we identify DEGs (fold change > = 2 and padj <= 0.05) [23].

### Using qRT-PCR to validate differentially expressed genes

To verify the gene expression data from RNA-Seq, we used qRT-PCR to quantify expression levels of the DEGs in response to salt stress. The qRT-PCR were performed according to the published studies [43, 44]. We use actin as a reference gene [45]. The relative expression level was calculated, based on the expression level of each gene without salt treatment. The primers are listed in Supplemental Table 5.

### Gene co-expression networks and gene ontology analyses

Using the WGCNA package in R, we identified gene co-expression based gene networks [24]. Cytoscape was then used to visualize the results [46]. The 21 RNA-Seq data mentioned above were used for co-expression analysis. Only genes with sum of expression levels are greater than 10 across the samples are used for the analysis. Regarding the parameters used, the best β (soft threshold power) value was set to 18 (after 20 iterations), the “deepSplit” value to 2, the “minModuleSize” value to 30, and the “mergeCutHeight” value to 0.15. The correlation coefficient is calculated using Pearson algorithm. The top 20% genes with the strongest weights were used to draw the co-expression networks.

Gene set enrichment analysis was performed using the clusterprofiler package in R [47]. We focused on biological processes. The *P*-value of each pathway was calculated and adjusted using the Benjamin-Hochberg method [48].

## Supplementary Information

**Additional file 1: Supplemental Table 1.** Annotations of bZIP protein sequence motifs.

**Additional file 2: Supplemental Table 2.** Segmentally duplicated bZIP gene pairs in poplar.

**Additional file 3: Supplemental Table 3.** List of syntenic gene pairs.

**Additional file 4: Supplemental Table 4.** Annotations of DEGs.

**Additional file 5: Supplemental Table 5**. Primer sequences.

**Additional file 6: Supplemental Data 1.** Pairwise gene expressions. C denotes without salt treatment. The DEGs were identified by use of the DESeq software and the thresholds were set at both fold change > = 2 and padj <= 0.05.

**Additional file 7: Supplemental Data 2.** DEGs in response to salt stress. L, S, and R denote leaves, stems, and roots, respectively. T and C represent with and without salt treatment, respectively. DEGs were identified by use of the DESeq software and the thresholds were set at both fold change > = 2 and padj <= 0.05.

**Additional file 8: Supplemental Data 3.** Co-expression gene sets.

## Data Availability

All data generated or analysed during this study are included in this published article and its supplementary information files. The raw sequencing data used during this study has been deposited in NCBI SRA with the accession number SRP267437.

## Abbreviations

DEGs: Differentially expressed genes
SAMS: S-Adenosyl-L-methionine synthetase
MCScanX: Multiple Collinearity Scan toolkit
Ka: Non-synonymous
Ks: Synonymous
WGCNA: Weighted Correlation Network Analysis
