# Supplementary material for: Genome-wide analysis and expression profile of the bZIP gene family in poplar
Source: BMC Plant Biol. 2021 Mar 1;21:122. doi: 10.1186/s12870-021-02879-w (PMC7919096; doi:10.1186/s12870-021-02879-w)
Supplement: Supplementary file 1 — Additional file 1: Supplemental Table 1. Annotations of bZIP protein sequence motifs. [file 12870_2021_2879_MOESM1_ESM.doc]

**Annotations of bZIP protein sequence motifs**

| Name | Sequence | Description of Smart | Description of Pfam |
| --- | --- | --- | --- |
| Motif 1  Motif 2  Motif 3  Motif 4  Motif 5  Motif 6  Motif 7  Motif 8  Motif 9  Motif 10  Motif 11  Motif 12  Motif 13  Motif 14  Motif 15  Motif 16  Motif 17  Motif 18  Motif 19  Motif 20 | IDEKRQRRMISNRESARRSRLRKQAYLQELESKVSKLREEN  DVFHLJSGMWKTPAERCFLWIGGFRPSELLKJLVNQLEPLTEQQLMGIYN  LEGFVRQADNLRQQTLQQMHRILTTRQAARALLAIGEYFSRLRALSSLWL  FDMEYARWLEEQHRHISELRTALNSHISDTELRIJVDNVMAHYDELFRLK  LSAQVTLLQRDTLGLTVENSELKLRLQAMEQQAQLRDALNEALKKEVERL  QQSSQQAEDALSQGMEALQQSLAETJASG  NQLGNLGKPLGSMNLDELLKNVWTAEETQ  NQJLTRJNVVSQHYDNVEAENSILRAEISELRHRLKSLNEI  KVPZRQPTLGEMTLEDFLVKAGVVREAT  LQRQGSLTLPRTLSKKTVDEVWKDIQQEK  QELQRARQQGJFJGSSGDQSH  QPQPLVRQSSLYSLTLDEVQ  HPYMWGPQHPMPPPYGPPYPY  LETQQLPLQKGTQVNPVSIPSQNPENWGETNMADASPRTDTSTDADTDDK  LLKNTRACTHTHTCNPPGPDYSHTHTCYHTHTKVJSSEEDD  SSGNVANYMGQMAMAMGKLGT  MYPHGVYAHPSIPPGSYPFSPFAMPSPNGIAEASGNTPGSMEADGRPSDA  NLNIGMDYWGAPASSTVPAIRGKVPSTPVAGGVVSTGSRDGVQSQIWLQD  MNSTSTQFVALGGMGIYDPFHQIGSWGEAFKDDGNLNTGPSTIVGNPA  TGKNNEHGKTTGVSANGAYSKSAESGSEGSSEGSDANSQSD | BRLZ domain  NO  Low complexity region  NO  Coiled coil region  NO  NO  Coiled coil region  NO  NO  NO  NO  NO  Low complexity region  Low complexity region  NO  NO  NO  NO  Low complexity region | bZIP_1  DOG1  NO  DOG1  NO  NO  NO  NO  NO  NO  NO  NO  NO  NO  NO  NO  NO  NO  NO  MFMR_assoc |
