# Supplementary material for: Genome-wide analysis and expression profile of the bZIP gene family in poplar
Source: BMC Plant Biol. 2021 Mar 1;21:122. doi: 10.1186/s12870-021-02879-w (PMC7919096; doi:10.1186/s12870-021-02879-w)
Supplement: Supplementary file 2 — Additional file 2: Supplemental Table 2. Segmentally duplicated bZIP gene pairs in poplar. [file 12870_2021_2879_MOESM2_ESM.doc]

Segmentally duplicated poplar bZIP gene pairs

| Gene ID | Gene ID | Duplication Type | Ka | Ks | Ka/Ks | Selection pressure |
| --- | --- | --- | --- | --- | --- | --- |
| Potri.001G136000.1  Potri.001G020200.1  Potri.001G029800.1  Potri.002G031900.1  Potri.002G045800.1  Potri.002G067400.1  Potri.002G069500.1  Potri.002G090700.1  Potri.002G115900.1  Potri.002G125400.1  Potri.002G167100.1  Potri.002G196200.1  Potri.004G140600.1  Potri.004G158200.1  Potri.004G163800.1  Potri.004G175200.1  Potri.004G111100.1  Potri.005G119300.1  Potri.005G126000.1  Potri.005G082000.1  Potri.005G053200.1  Potri.006G058800.1  Potri.006G025800.1  Potri.006G034500.1  Potri.007G130800.1  Potri.006G251800.1  Potri.008G118300.1  Potri.008G106700.1  Potri.008G010800.1  Potri.013G156900.1  Potri.004G203400.3 | Potri.003G097600.1  Potri.003G204400.1  Potri.003G194600.1  Potri.005G231300.1  Potri.005G217500.1  Potri.005G192900.1  Potri.005G190700.1  Potri.005G170500.1  Potri.014G013400.1  Potri.014G028200.1  Potri.014G094200.1  Potri.014G120800.1  Potri.009G101200.1  Potri.009G119700.1  Potri.009G125400.1  Potri.009G134900.1  Potri.017G106700.1  Potri.007G019900.1  Potri.007G029400.1  Potri.007G085700.1  Potri.013G040700.1  Potri.016G049200.1  Potri.016G024000.1  Potri.016G032400.1  Potri.017G027400.1  Potri.018G029500.1  Potri.010G128000.1  Potri.010G142900.1  Potri.010G248100.1  Potri.019G130000.1  Potri.009G164300.1 | Segmental duplication  Segmental duplication  Segmental duplication  Segmental duplication  Segmental duplication  Segmental duplication  Segmental duplication  Segmental duplication  Segmental duplication  Segmental duplication  Segmental duplication  Segmental duplication  Segmental duplication  Segmental duplication  Segmental duplication  Segmental duplication  Segmental duplication  Segmental duplication  Segmental duplication  Segmental duplication  Segmental duplication  Segmental duplication  Segmental duplication  Segmental duplication  Segmental duplication  Segmental duplication  Segmental duplication  Segmental duplication  Segmental duplication  Segmental duplication  Segmental duplication | 0.036872  0.113276  0.050496  0.045350  0.101833  0.048402  0.098006  0.038273  0.141557  0.082021  0.120244  0.089554  0.118930  0.072828  0.047438  0.106550  0.061225  0.117824  0.092654  0.062915  0.098942  0.043850  0.077652  0.090779  0.048613  0.121870  0.099614  0.119157  0.039077  0.065637  0.033192 | 0.239559  0.298376  0.191252  0.260521  0.229956  0.305090  0.428869  0.208387  0.356568  0.303873  0.299897  0.356669  0.285285  0.581923  0.309955  0.333225  0.302716  0.283616  0.342106  0.249607  0.245562  0.209488  0.335574  0.321298  0.244189  0.370668  0.300027  0.332120  0.118435  0.277325  0.282440 | 0.153917  0.379641  0.264028  0.174076  0.442837  0.158648  0.228521  0.183662  0.396998  0.269918  0.400952  0.251084  0.416883  0.125150  0.153047  0.319752  0.202251  0.415437  0.270834  0.252055  0.402920  0.209320  0.231400  0.282538  0.199080  0.328786  0.332015  0.358778  0.329942  0.236679  0.117518 | Purifying selection  Purifying selection  Purifying selection  Purifying selection  Purifying selection  Purifying selection  Purifying selection  Purifying selection  Purifying selection  Purifying selection  Purifying selection  Purifying selection  Purifying selection  Purifying selection  Purifying selection  Purifying selection  Purifying selection  Purifying selection  Purifying selection  Purifying selection  Purifying selection  Purifying selection  Purifying selection  Purifying selection  Purifying selection  Purifying selection  Purifying selection  Purifying selection  Purifying selection  Purifying selection  Purifying selection |
