# Supplementary material for: Genome-wide analysis and expression profile of the bZIP gene family in poplar
Source: BMC Plant Biol. 2021 Mar 1;21:122. doi: 10.1186/s12870-021-02879-w (PMC7919096; doi:10.1186/s12870-021-02879-w)
Supplement: Supplementary file 3 — Additional file 3: Supplemental Table 3. List of syntenic gene pairs. [file 12870_2021_2879_MOESM3_ESM.doc]

Syntenic gene pairs (*P. trichocarpa* and *A. thaliana*)

| Gene ID | Gene ID | Ka | | Ks | Ka/Ks | | Selection pressure |
| --- | --- | --- | --- | --- | --- | --- | --- |
| Potri.002G090700.1  Potri.002G031900.1  Potri.002G167100.1  Potri.002G167100.1  Potri.004G163800.1  Potri.004G140600.1  Potri.004G163800.1  Potri.005G170500.1  Potri.005G119300.1  Potri.009G125400.1  Potri.009G101200.1  Potri.009G119700.1  Potri.009G134900.1  Potri.009G125400.1  Potri.010G128000.1  Potri.014G094200.1 | AT1G77920.1  AT1G75390.1  AT2G46270.1  AT4G01120.1  AT4G38900.1  AT3G19290.3  AT2G21230.3  AT1G77920.1  AT2G18160.1  AT4G38900.1  AT3G19290.3  AT4G34590.1  AT4G35040.1  AT2G21230.3  AT1G68640.1  AT4G01120.1 | | 0.236881  0.375891  0.318763  0.331276  0.244923  0.315171  0.301231  0.218191  0.446616  0.251799  0.360071  0.373199  0.266846  0.319178  0.403096  0.379044 | | --- | | 2.159256  NaN  1.771746  1.196673  NaN  2.852188  2.671067  2.539496  2.005611  NaN  2.134035  NaN  2.267014  2.041082  1.441812  1.210825 | | 0.109705  NaN  0.179915  0.276831  NaN  0.110502  0.112775  0.085919  0.222683  NaN  0.168728  NaN  0.117708  0.156377  0.279576  0.313046 | Purifying selection  No  Purifying selection  Purifying selection  No  Purifying selection  Purifying selection  Purifying selection  Purifying selection  No  Purifying selection  No  Purifying selection  Purifying selection  Purifying selection  Purifying selection | |

Syntenic gene pairs (*P. trichocarpa* and *G. max*)

| Gene ID | Gene ID | Ka | Ks | Ka/Ks | Selection pressure | |
| --- | --- | --- | --- | --- | --- | --- |
| Potri.001G136000.1  Potri.001G136000.1  Potri.001G029800.1  Potri.001G029800.1  Potri.002G196200.1  Potri.002G031900.1  Potri.002G196200.1  Potri.002G125400.1  Potri.002G090700.1  Potri.002G069500.1  Potri.002G167100.1  Potri.002G115900.1  Potri.002G125400.1  Potri.002G090700.1  Potri.002G031900.1  Potri.002G196200.1  Potri.002G069500.1  Potri.002G167100.1  Potri.002G090700.1  Potri.002G090800.1  Potri.002G031900.1  Potri.002G090700.1  Potri.002G090800.1  Potri.002G031900.1  Potri.002G167100.1  Potri.002G069500.1  Potri.002G167100.1  Potri.002G196200.1  Potri.003G097600.1  Potri.003G097600.1  Potri.003G194600.1  Potri.003G194600.1  Potri.004G140600.1  Potri.004G111100.1  Potri.004G140600.1  Potri.004G158200.1  Potri.004G158200.1  Potri.004G163800.1  Potri.004G158200.1  Potri.004G158200.1  Potri.004G111100.1  Potri.005G126000.1  Potri.005G170500.1  Potri.005G082000.1  Potri.005G231300.1  Potri.005G170500.1  Potri.005G053200.1  Potri.005G190700.1  Potri.005G126000.1  Potri.005G126000.1  Potri.005G082000.1  Potri.005G190700.1  Potri.005G053200.1  Potri.005G082000.1  Potri.005G231300.1  Potri.005G170500.1  Potri.005G231300.1  Potri.005G170500.1  Potri.005G082000.1  Potri.005G053200.1  Potri.005G126000.1  Potri.005G190700.1  Potri.005G231300.1  Potri.006G058800.1  Potri.006G034500.1  Potri.006G039000.1  Potri.006G114600.1  Potri.006G114600.1  Potri.006G034500.1  Potri.006G058800.1  Potri.007G085700.1  Potri.007G130800.1  Potri.007G130800.1  Potri.007G006900.1  Potri.007G029400.1  Potri.007G130800.1  Potri.007G085700.1  Potri.007G029400.1  Potri.007G006900.1  Potri.007G085700.1  Potri.007G029400.1  Potri.007G085700.1  Potri.007G029400.1  Potri.007G130800.1  Potri.007G006900.1  Potri.008G118300.1  Potri.008G118300.1  Potri.008G118300.1  Potri.009G101200.1  Potri.009G119700.1  Potri.009G119700.1  Potri.009G125400.1  Potri.009G119700.1  Potri.009G119700.1  Potri.009G101200.1  Potri.010G128000.1  Potri.010G135200.1  Potri.010G128000.1  Potri.010G128000.1  Potri.010G128000.1  Potri.013G040700.1  Potri.013G156900.1  Potri.013G040700.1  Potri.013G040700.1  Potri.013G156900.1  Potri.013G156900.1  Potri.014G120800.1  Potri.014G120800.1  Potri.014G094200.1  Potri.014G120800.1  Potri.014G028200.1  Potri.014G013400.1  Potri.014G028200.1  Potri.014G120800.1  Potri.014G094200.1  Potri.014G094200.1  Potri.016G032400.1  Potri.016G036500.1  Potri.016G049200.1  Potri.016G032400.1  Potri.016G036500.1  Potri.016G049200.1  Potri.017G106700.1  Potri.017G106700.1  Potri.019G130000.1  Potri.019G130000.1  Potri.019G130000.1 | Glyma.01G013600.1  Glyma.09G208500.1  Glyma.13G193700.1  Glyma.15G232000.1  Glyma.02G012700.1  Glyma.04G029600.1  Glyma.03G219300.1  Glyma.04G039300.1  Glyma.04G254800.1  Glyma.02G082800.1  Glyma.03G255000.1  Glyma.06G048500.1  Glyma.06G040400.1  Glyma.06G107300.1  Glyma.06G029600.1  Glyma.10G013300.1  Glyma.10G226000.1  Glyma.07G060400.1  Glyma.14G167000.1  Glyma.14G167100.1  Glyma.14G071400.1  Glyma.13G085100.1  Glyma.13G085300.1  Glyma.17G253200.1  Glyma.16G029000.1  Glyma.16G168400.1  Glyma.19G252600.1  Glyma.19G216200.1  Glyma.01G013600.1  Glyma.09G208500.1  Glyma.15G232000.1  Glyma.13G193700.1  Glyma.07G213100.1  Glyma.08G254400.1  Glyma.02G131700.1  Glyma.06G010200.1  Glyma.04G010300.1  Glyma.12G036400.1  Glyma.12G040600.1  Glyma.11G114800.1  Glyma.18G277100.1  Glyma.01G177400.1  Glyma.06G107300.1  Glyma.05G182500.1  Glyma.04G029600.1  Glyma.04G254800.1  Glyma.03G247100.1  Glyma.02G082800.1  Glyma.02G058800.1  Glyma.11G065000.1  Glyma.11G236300.1  Glyma.10G226000.1  Glyma.10G162100.1  Glyma.08G140100.1  Glyma.06G029600.1  Glyma.14G167000.1  Glyma.14G071400.1  Glyma.13G085100.1  Glyma.18G020900.1  Glyma.19G244800.1  Glyma.16G141500.1  Glyma.16G168400.1  Glyma.17G253200.1  Glyma.03G142400.1  Glyma.03G123200.1  Glyma.03G128200.1  Glyma.03G193300.1  Glyma.19G193400.1  Glyma.19G126800.1  Glyma.19G145300.1  Glyma.08G140100.1  Glyma.13G345200.1  Glyma.12G051200.1  Glyma.11G045900.1  Glyma.11G065000.1  Glyma.11G126600.1  Glyma.11G236300.1  Glyma.01G177400.1  Glyma.05G108200.1  Glyma.05G182500.1  Glyma.02G058800.1  Glyma.18G020900.1  Glyma.16G141500.1  Glyma.15G029100.1  Glyma.17G158900.1  Glyma.01G084200.1  Glyma.20G113600.1  Glyma.10G276100.1  Glyma.02G131700.1  Glyma.04G010300.1  Glyma.12G040600.1  Glyma.12G036400.1  Glyma.11G114800.1  Glyma.06G010200.1  Glyma.07G213100.1  Glyma.10G276100.1  Glyma.02G126100.1  Glyma.02G097900.1  Glyma.01G084200.1  Glyma.20G113600.1  Glyma.03G247100.1  Glyma.02G236200.1  Glyma.10G162100.1  Glyma.19G244800.1  Glyma.18G052500.1  Glyma.14G204100.1  Glyma.19G216200.1  Glyma.02G012700.1  Glyma.03G255000.1  Glyma.03G219300.1  Glyma.04G039300.1  Glyma.06G048500.1  Glyma.06G040400.1  Glyma.10G013300.1  Glyma.16G029000.1  Glyma.19G252600.1  Glyma.03G123200.1  Glyma.03G127600.1  Glyma.03G142400.1  Glyma.19G126800.1  Glyma.19G130200.1  Glyma.19G145300.1  Glyma.19G037900.1  Glyma.13G050700.1  Glyma.02G236200.1  Glyma.14G204100.1  Glyma.18G052500.1 | | 0.113778  0.120434  0.158970  0.178465  0.415927  0.336753  0.322861  0.223330  0.257114  0.313895  0.194887  0.342423  0.258004  0.250389  0.344101  0.343945  0.596881  0.235156  0.261738  0.499504  0.316859  0.264270  0.511862  0.356504  0.229226  0.322875  0.203260  0.346269  0.120435  0.121361  0.185183  0.158607  0.282609  0.217725  0.306732  0.238352  0.198079  0.180655  0.154192  0.156907  0.214631  0.221910  0.255356  0.239401  0.337931  0.261561  0.298511  0.307435  0.202359  0.229419  0.187066  0.673876  0.267608  0.224937  0.313730  0.271301  0.361670  0.271802  0.185135  0.260176  0.199870  0.373711  0.338892  0.146700  0.423228  0.131409  0.452276  0.480212  0.422109  0.148186  0.213422  0.147498  0.320972  0.298974  0.245269  0.279027  0.172459  0.219346  0.388262  0.229272  0.196601  0.171288  0.195525  0.130860  0.338415  0.180957  0.180763  0.182262  0.346273  0.192610  0.155372  0.172045  0.159773  0.219740  0.305050  0.408106  0.224772  0.327431  0.355259  0.393202  0.249862  0.197936  0.256911  0.247961  0.197282  0.204208  0.376295  0.415003  0.195055  0.351421  0.246971  0.348925  0.292765  0.371387  0.244887  0.199927  0.394213  0.131577  0.107364  0.375119  0.125465  0.111476  0.194720  0.224382  0.192242  0.185138  0.211747 | | --- | | 0.920609  0.835074  1.227478  1.223608  NaN  NaN  NaN  1.973005  1.001216  1.270236  0.981157  1.549849  1.501677  1.080281  NaN  NaN  2.555645  0.976465  0.829086  1.141333  1.501623  0.877629  1.263994  1.425429  1.070892  1.280534  0.960486  NaN  0.883164  0.919184  1.296712  1.326713  1.371562  1.482109  1.414035  1.407672  1.167914  2.756233  1.746677  2.129865  1.823209  0.959233  1.151299  1.170751  NaN  1.161314  0.962167  1.489437  0.944704  1.024871  1.042710  1.533217  1.133627  1.138893  NaN  0.975028  2.310373  1.053981  1.021801  1.046963  0.930468  1.133555  1.634783  1.385887  1.333393  1.317659  1.200931  1.045815  1.436614  1.172266  1.065077  1.659993  2.439892  2.810319  1.086635  2.149232  0.951508  1.115610  2.151454  1.129949  0.950118  0.926165  0.895310  1.695247  2.582371  1.248092  1.251932  1.181292  1.675785  1.524221  2.015209  2.741464  2.273474  1.547770  1.634987  1.390060  1.175018  0.993874  0.947159  1.513892  1.026307  NaN  1.167945  1.079236  NaN  NaN  NaN  NaN  1.138727  NaN  2.267348  1.695303  1.532487  NaN  1.066327  1.127585  1.322335  1.101081  1.340945  1.231706  1.033008  1.083085  NaN  NaN  4.094862  NaN  NaN | 0.123590  0.144220  0.129509  0.145852  NaN  NaN  NaN  0.113193  0.256802  0.247115  0.198630  0.220940  0.171810  0.231781  NaN  NaN  0.233554  0.240824  0.315695  0.437650  0.211011  0.301118  0.404956  0.250103  0.214051  0.252141  0.211622  NaN  0.136367  0.132031  0.142810  0.119549  0.206049  0.146902  0.216920  0.169323  0.169600  0.065544  0.088277  0.073670  0.117722  0.231341  0.221798  0.204485  NaN  0.225229  0.310248  0.206410  0.214204  0.223852  0.179403  0.439518  0.236064  0.197505  NaN  0.278249  0.156542  0.257881  0.181185  0.248505  0.214805  0.329681  0.207301  0.105853  0.317407  0.099729  0.376605  0.459175  0.293822  0.126410  0.200382  0.088854  0.131552  0.106384  0.225714  0.129826  0.181248  0.196616  0.180465  0.202905  0.206923  0.184943  0.218388  0.077192  0.131048  0.144987  0.144387  0.154290  0.206633  0.126366  0.077100  0.062757  0.070277  0.141972  0.186576  0.293589  0.191292  0.329449  0.375079  0.259729  0.243457  NaN  0.219969  0.229756  NaN  NaN  NaN  NaN  0.171292  NaN  0.108925  0.205819  0.191039  NaN  0.229655  0.177306  0.298119  0.119498  0.080066  0.304552  0.121456  0.102925  NaN  NaN  0.046947  NaN  NaN | | Purifying selection  Purifying selection  Purifying selection  Purifying selection  No  No  No  Purifying selection  Purifying selection  Purifying selection  Purifying selection  Purifying selection  Purifying selection  Purifying selection  No  No  Purifying selection  Purifying selection  Purifying selection  Purifying selection  Purifying selection  Purifying selection  Purifying selection  Purifying selection  Purifying selection  Purifying selection  Purifying selection  No  Purifying selection  Purifying selection  Purifying selection  Purifying selection  Purifying selection  Purifying selection  Purifying selection  Purifying selection  Purifying selection  Purifying selection  Purifying selection  Purifying selection  Purifying selection  Purifying selection  Purifying selection  Purifying selection  No  Purifying selection  Purifying selection  Purifying selection  Purifying selection  Purifying selection  Purifying selection  Purifying selection  Purifying selection  Purifying selection  No  Purifying selection  Purifying selection  Purifying selection  Purifying selection  Purifying selection  Purifying selection  Purifying selection  Purifying selection  Purifying selection  Purifying selection  Purifying selection  Purifying selection  Purifying selection  Purifying selection  Purifying selection  Purifying selection  Purifying selection  Purifying selection  Purifying selection  Purifying selection  Purifying selection  Purifying selection  Purifying selection  Purifying selection  Purifying selection  Purifying selection  Purifying selection  Purifying selection  Purifying selection  Purifying selection  Purifying selection  Purifying selection  Purifying selection  Purifying selection  Purifying selection  Purifying selection  Purifying selection  Purifying selection  Purifying selection  Purifying selection  Purifying selection  Purifying selection  Purifying selection  Purifying selection  Purifying selection  Purifying selection  No  Purifying selection  Purifying selection  No  No  No  No  Purifying selection  No  Purifying selection  Purifying selection  Purifying selection  No  Purifying selection  Purifying selection  Purifying selection  Purifying selection  Purifying selection  Purifying selection  Purifying selection  Purifying selection  No  No  Purifying selection  No  No |

Syntenic gene pairs (*P. trichocarpa* and *S. lycopersicum*)

| Gene ID | Gene ID | Ka | Ks | Ka/Ks | Selection pressure | |
| --- | --- | --- | --- | --- | --- | --- |
| Potri.002G196200.1  Potri.002G167100.1  Potri.002G031900.1  Potri.002G067400.1  Potri.002G069500.1  Potri.002G125400.1  Potri.004G158200.1  Potri.004G163800.1  Potri.004G175200.1  Potri.005G119300.1  Potri.005G126000.1  Potri.005G053200.1  Potri.005G082000.1  Potri.005G231300.1  Potri.005G190700.1  Potri.005G192900.1  Potri.006G114600.1  Potri.007G006900.1  Potri.007G085700.1  Potri.007G019900.1  Potri.007G029400.1  Potri.008G118300.1  Potri.009G119700.1  Potri.009G125400.1  Potri.009G134900.1  Potri.009G101200.1  Potri.009G134900.1  Potri.010G128000.1  Potri.013G040700.1  Potri.014G120800.1  Potri.014G094200.1  Potri.014G028200.1  Potri.016G049200.1  Potri.017G106700.1 | Solyc01g079480.2.1  Solyc01g095460.2.1  Solyc04g080740.1.1  Solyc04g071510.2.1  Solyc04g071160.2.1  Solyc04g078840.2.1  Solyc01g109880.2.1  Solyc01g110480.2.1  Solyc01g111580.2.1  Solyc02g084860.1.1  Solyc02g085610.2.1  Solyc01g097330.2.1  Solyc04g054320.2.1  Solyc04g080740.1.1  Solyc04g071160.2.1  Solyc04g071510.2.1  Solyc10g083380.1.1  Solyc02g092090.1.1  Solyc04g054320.2.1  Solyc02g084860.1.1  Solyc02g085610.2.1  Solyc05g009660.2.1  Solyc01g109880.2.1  Solyc01g110480.2.1  Solyc01g111580.2.1  Solyc01g108080.2.1  Solyc10g055550.1.1  Solyc05g009660.2.1  Solyc01g097330.2.1  Solyc01g079480.2.1  Solyc01g095460.2.1  Solyc04g078840.2.1  Solyc10g080410.1.1  Solyc02g089420.1.1 | | 0.350569  0.299609  0.228185  0.379046  0.291464  0.243977  0.213298  0.159143  0.251639  0.314578  0.233624  0.309241  0.258678  0.218465  0.337466  0.360316  0.745941  0.347093  0.248039  0.296567  0.242659  0.164683  0.198530  0.161627  0.245397  0.319549  0.349887  0.315328  0.280164  0.430160  0.317974  0.255238  0.204513  0.197311 | | --- | | 3.186371  1.052630  2.311617  2.069556  1.612042  2.385780  NaN  2.016645  2.411819  1.863680  1.283587  1.467460  1.557857  2.042488  1.871330  1.804435  2.664252  1.382538  1.419499  1.732383  1.263103  1.450485  1.818405  1.796462  2.180104  1.346007  4.120536  1.585350  1.249983  3.442196  1.397729  1.640650  1.315067  1.251618 | 0.110021  0.284629  0.098712  0.183153  0.180804  0.102263  NaN  0.078915  0.104336  0.168794  0.182008  0.210732  0.166047  0.106960  0.180335  0.199684  0.279982  0.251055  0.174737  0.171190  0.192113  0.113536  0.109178  0.089969  0.112562  0.237405  0.084913  0.198901  0.224134  0.124967  0.227494  0.155571  0.155515  0.157645 | | Purifying selection  Purifying selection  Purifying selection  Purifying selection  Purifying selection  Purifying selection  No  Purifying selection  Purifying selection  Purifying selection  Purifying selection  Purifying selection  Purifying selection  Purifying selection  Purifying selection  Purifying selection  Purifying selection  Purifying selection  Purifying selection  Purifying selection  Purifying selection  Purifying selection  Purifying selection  Purifying selection  Purifying selection  Purifying selection  Purifying selection  Purifying selection  Purifying selection  Purifying selection  Purifying selection  Purifying selection  Purifying selection  Purifying selection |

Syntenic gene pairs (*P. trichocarpa* and *A. comosus*)

| Gene ID | Gene ID | Ka | | Ks | Ka/Ks | | Selection pressure |
| --- | --- | --- | --- | --- | --- | --- | --- |
| Potri.002G196200.1  Potri.002G090700.1  Potri.002G196200.1  Potri.005G170500.1  Potri.006G251800.1  Potri.009G134900.1  Potri.014G120800.1  Potri.014G120800.1  Potri.018G029500.1 | Aco002544.1  Aco001167.1  Aco001785.1  Aco001167.1  Aco014284.1  Aco013098.1  Aco001785.1  Aco002544.1  Aco014284.1 | | 0.382710  0.232950  0.738861  0.235379  0.443651  0.403086  0.768787  0.413769  0.258825 | | --- | | NaN  1.938716  2.673392  2.474406  NaN  NaN  2.007381  2.001250  NaN | | NaN  0.120157  0.276376  0.095125  NaN  NaN  0.382980  0.206755  NaN | No  Purifying selection  Purifying selection  Purifying selection  No  No  Purifying selection  Purifying selection  No | |

Syntenic gene pairs (*P. trichocarpa* and *O. sativa*)

| Gene ID | Gene ID | Ka | | Ks | Ka/Ks | | Selection pressure |
| --- | --- | --- | --- | --- | --- | --- | --- |
| NO |  | |  | | --- | |  | |  |  | |

Syntenic gene pairs (*P. trichocarpa* and *Z. mays*)

| Gene ID | Gene ID | Ka | | Ks | Ka/Ks | | Selection pressure |
| --- | --- | --- | --- | --- | --- | --- | --- |
| NO |  | |  | | --- | |  | |  |  | |
