# Supplementary material for: Genome-wide analysis and expression profile of the bZIP gene family in poplar
Source: BMC Plant Biol. 2021 Mar 1;21:122. doi: 10.1186/s12870-021-02879-w (PMC7919096; doi:10.1186/s12870-021-02879-w)
Supplement: Supplementary file 4 — Additional file 4: Supplemental Table 4. Annotations of DEGs. [file 12870_2021_2879_MOESM4_ESM.doc]

Annotations of differentially expressed genes

|  | ID | Best-hit-arabi-name |
| --- | --- | --- |
| Gene differently expressed in leaf, root, and stem without salt treatment | Potri.008G113400.1  Potri.005G082000.1  Potri.009G164300.1  Potri.007G085700.1  Potri.007G019900.1 | AT3G30530.1  AT5G65210.1  AT1G08320.1  AT5G65210.1  AT1G75390.1 |
| Differential expression genes in response to salinity | Potri.005G243400.1  Potri.019G091900.1  Potri.001G374200.1  Potri.013G156900.1  Potri.004G158200.1  Potri.005G053200.1  Potri.005G231300.1  Potri.019G130000.1  Potri.005G192900.1  Potri.002G167100.1  Potri.004G140600.1  Potri.014G120800.1  Potri.002G125400.1  Potri.014G094200.1  Potri.007G085700.1  Potri.002G090700.1  Potri.010G142900.1  Potri.008G106700.1  Potri.014G028200.1  Potri.016G024000.1  Potri.013G091400.1  Potri.003G204400.1  Potri.004G203400.3  Potri.001G020200.1  Potri.010G135200.1  Potri.003G194600.1  Potri.005G170500.1  Potri.009G164300.1  Potri.005G082000.1  Potri.006G058800.1  Potri.009G119700.1  Potri.005G119300.1  Potri.006G034500.1  Potri.016G032400.1  Potri.014G007100.1  Potri.014G013400.1  Potri.002G115900.1  Potri.002G196200.1  Potri.007G006900.1  Potri.006G083000.1  Potri.002G090800.1  Potri.018G029500.1  Potri.005G126000.1  Potri.008G113400.1  Potri.009G101200.1 | AT4G35900.1  AT3G58120.1  AT3G58120.1  AT2G40620.1  AT1G75390.1  AT5G28770.2  AT1G75390.1  AT2G40620.1  AT5G44080.1  AT2G46270.1  AT4G34000.1  AT3G62420.1  AT1G45249.1  AT2G46270.1  AT5G65210.1  AT1G77920.1  AT5G49450.1  AT3G62420.1  AT1G45249.1  AT3G56850.1  AT2G40620.1  AT2G16770.1  AT1G08320.1  AT4G35040.1  AT3G30530.1  AT3G12250.1  AT1G77920.1  AT1G08320.1  AT5G65210.1  AT5G06839.3  AT1G75390.1  AT1G75390.1  AT2G40950.1  AT2G40950.1  AT2G22850.1  AT1G59530.1  AT1G59530.1  AT3G62420.1  AT3G49760.1  AT2G36270.1  AT1G49720.1  AT5G11260.1  AT4G36730.1  AT3G30530.1  AT1G45249.1 |
