# Supplementary material for: Genome-wide analysis and expression profile of the bZIP gene family in poplar
Source: BMC Plant Biol. 2021 Mar 1;21:122. doi: 10.1186/s12870-021-02879-w (PMC7919096; doi:10.1186/s12870-021-02879-w)
Supplement: Supplementary file 5 — Additional file 5: Supplemental Table 5. Primer sequences. [file 12870_2021_2879_MOESM5_ESM.doc]

Primer sequences for qRT-PCR

| ID | 5’ primers | 3’ primers |
| --- | --- | --- |
| JM986590 (*ACT*) | ACCCTCCAATCCAGACACTG | TTGCTGACCGTATGAGCAAG |
| Potri.010G142900.1  Potri.009G101200.1  Potri.009G119700.1  Potri.004G158200.1  Potri.004G140600.1  Potri.013G156900.1  Potri.018G029500.1  Potri.007G085700.1  Potri.002G196200.1  Potri.002G125400.1  Potri.002G167100.1  Potri.002G090700.1  Potri.002G090800.1  Potri.019G091900.1  Potri.019G130000.1  Potri.005G231300.1  Potri.005G119300.1  Potri.005G053200.1  Potri.005G192900.1  Potri.014G120800.1  Potri.014G028200.1  Potri.008G106700.1  Potri.008G113400.1 | TTGACGGATGAAAAGTTGAAGC  GGACTCTCATTGGGACTTCAAG  CAAGCATCTTTAATGAGCCAGC  GAATGAGATAATCGGTTCCTTG  GGACTCTCAATGGGACTACAAG  GACATGACAATGATGATGATGG  GTCAAAGAAGGTATGGAGAGTG  CGTGTAGAGAGTGACATGAGCC  GCTGCGGTCGTTGAACTCTGTG  GGGTCAATGAACATGGATGAG  TGCCACAGCCTATGATGCCACC  CTAGTTGAGAATGGCTTGAATC  GGTGCCATCAAATGGAGCCCAG  GGACACATAGGAGGTCGGTGAG  ACCACCTGCTTCTTCGATGCTC  CTATATCAACACGAGTAATGGG  GGCTGAGCTTACCCACAGATTG  GAACCGGTCTGAATCTGAATGG  TTCGTCGAAGGTGATGGCGTCG  GCTGTGGAATTGACTGAGAGGC  GCAACCTCTGCTGGGGTACAAG  GGTTATGAAGTCCCTGAATCTG  GCACCTGATCAAAGCCTGATTC | CATGGGTTCAAAAATCGGTCTG  CATTGCTTGGGTCTCTTCAGCA  CTGCAGAGGCACTTATCGGCTG  CAGTAATGAAACATCTCTGCAG  GTACACTGAAGACTGGCGGATC  CCTGTTGAAGACCCACCATTAC  GCGTTTGAACCGGTCTCTCTAC  CGATTTCCACAAGCCAGACATC  CCACGGCTTCAACAAAGGATCG  CTCATCCACCGTCCTCTGACTC  GATGGGATACAATTGGAACAGC  GGTTACAGACATCCGCAAGCTG  GCTTCCTTACATTTCCGATCTG  CCACGGCATTGGAGATATCATC  CATTGATTTGATCTGATGGGTG  CAATACTGAAACAAATCTGGGG  GGCAACAGCATCCTCGGGTTC  GATGAAGCAGCAGTGGCACAGG  CGTCTACTGTCATTGTCGATTC  GGTATTTCAGGTATCTCCACGG  CATCCACCGTCTTCAGGCTCAG  GAATTTGAACATCCCAGAAGC  GTTGTTGCTGAGACTTGAGGAC |
